# A Comprehensive Review Exploring the Protective Role of Specific Commensal Gut Bacteria against *Salmonella*

**DOI:** 10.3390/pathogens13080642

**Published:** 2024-07-31

**Authors:** Saloni Singh, Ok Kyung Koo

**Affiliations:** Department of Food Science & Technology, Chungnam National University, Daejeon 34134, Republic of Korea; salamoniasingh12@gmail.com

**Keywords:** bacteria interaction, dysbiosis, gut bacteria, pathogen defense, *Salmonella*

## Abstract

Gut microbiota is a diverse community of microorganisms that constantly work to protect the gut against pathogens. *Salmonella* stands out as a notorious foodborne pathogen that interacts with gut microbes, causing an imbalance in the overall composition of microbiota and leading to dysbiosis. This review focuses on the interactions between *Salmonella* and the key commensal bacteria such as *E. coli*, *Lactobacillus*, *Clostridium*, *Akkermansia*, and *Bacteroides*. The review highlights the role of these gut bacteria and their synergy in combating *Salmonella* through several mechanistic interactions. These include the production of siderophores, which compete with *Salmonella* for essential iron; the synthesis of short-chain fatty acids (SCFAs), which exert antimicrobial effects and modulate the gut environment; the secretion of bacteriocins, which directly inhibit *Salmonella* growth; and the modulation of cytokine responses, which influences the host’s immune reaction to infection. While much research has explored *Salmonella*, this review aims to better understand how specific gut bacteria engage with the pathogen, revealing distinct defense mechanisms tailored to each species and how their synergy may lead to enhanced protection against *Salmonella*. Furthermore, the combination of these commensal bacteria could offer promising avenues for bacteria-mediated therapy during *Salmonella*-induced gut infections in the future.

## 1. Introduction

*Salmonella* is a common bacterial cause of diarrheal infections, accounting for over 150 million cases and 60,000 deaths globally each year [1]. Most of these illnesses stem from contaminated food or water sources, highlighting the critical role of food safety in preventing *Salmonella* infections. Ingested *Salmonella* moves through the stomach and reaches the small intestine, where it encounters the intestinal epithelial cells. *Salmonella* adhesins bind to host cell receptors, resulting in membrane ruffling and bacterial internalization into the host cell cytoplasm [2]. Once within these cells, it can replicate and spread to adjacent ones using mechanisms such as actin-based motility and the formation of *Salmonella*-induced filaments [3]. Intracellular replication allows the evasion of host immune responses and the establishment of gut mucosa by *Salmonella* as, once the infection is established, the host begins an immune response that involves the recruitment of immune cells like neutrophils or macrophages at the site of infection [4]. The role played by these immune cells is to fight against infections through the phagocytosis of *Salmonella* and the production of proinflammatory cytokines. Phagocytosing *Salmonella* is one of the ways in which these cells try to hold back the infection while, at the same time, producing proinflammatory cytokines. However, *Salmonella* has other ways to protect itself from the host immune response such as inhibiting phagolysosome fusion and modulating host cell signaling [5,6]. In effect, *Salmonella* might as well invade this barrier and then spread towards systemic sites where it causes systemic salmonellosis, known for exhibiting symptoms such as fever and diarrhea or, at worse, septicemia [4,7]. An end result of such bacterial virulence factors, together with the attendant host immune responses, is that *Salmonella* gains entry into epithelial cells in the gut and commences intracellular replication, thus causing dissemination.

Maintaining a stable intestinal microbiota is vital in protecting against infection through various mechanisms [8,9]. Commensal bacteria found within the gut flora compete with pathogens for sites of colonization and nutrients, thus impeding their growth or establishment. In addition, these commensal microorganisms manufacture antimicrobial substances, including bacteriocins and short-chain fatty acids (SCFAs) that kill invasive pathogenic microorganisms [8,9]. Apart from that, a rich microbial profile plays a vital role in the maturation and functioning of the host immune system. Commensal bacteria interact with epithelial cells in the intestine and cells in the immune system, producing immunoglobulins like secretory IgA, which can bind pathogens or neutralize them on the epithelium lining of the gut. In addition to this, short-chain fatty acids produced by constituents of gut microflora like butyric acid are essential for the preservation of intestinal barrier function, hence preventing translocation across the epithelium lining of the bowel because they enhance mucin as well as tight junction protein synthesis [8,9,10].

Commensal bacteria, including *E. coli*, *Clostridium*, *Lactobacillus*, *Bacteroides*, and *Akkermansia*, are mainly involved in nutrient competition, antimicrobial substances, gut barrier integrity, immune system modulation, and SCFA production [11,12,13,14]. While previous research has explored the mechanisms, a comprehensive understanding of how specific commensal strains inhibit *Salmonella* can provide valuable insights for developing targeted probiotic interventions to combat infections. Therefore, this investigation allows for a more manageable and detailed analysis of their mechanisms of action compared to studying the entire gut microbiota. Moreover, identifying key commensal strains involved in *Salmonella* inhibition enables the development of more precise and effective strategies for promoting gut health and preventing enteric infections. Through delving into the intricate crosstalk between *Salmonella* and these beneficial gut bacteria, this review aims to provide a comprehensive understanding of host defense mechanisms against *Salmonella* infection while identifying promising avenues for future research and therapeutic interventions.

## 2. Ecocompetiton

*Salmonella* and other bacteria compete intensely for various nutrient niches in the gut ecosystem (ecocompetition). This interaction depends on the availability of nutrients, such as iron and oxygen, with different microbial species competing for these essential resources. Dietary composition significantly influences these niches, determining which microbial populations can establish and maintain themselves. The inherent resilience of the gut microbiota, supported by diet and nutrient competition, often inhibits the successful colonization of *Salmonella* in the intestinal lining, thereby safeguarding the host from potential infections. 

### 2.1. Siderophores 

Iron plays a multifaceted role in bacterial systems as a pivotal cofactor for iron-containing proteins. Its involvement extends across vital functions, including redox reactions, metabolic pathways, and the intricate mechanisms of the electron transport chain [15]. *Salmonella* uses different strategies to compete for iron-binding molecules called siderophores in the gut environment. Iron is limited in the gut due to iron-scavenging proteins from the host and competition from other gut bacteria. *Salmonella* produces its siderophores, which allow it to scavenge iron from the gut and outcompete other gut microbes for this essential nutrient [16]. *Salmonella* also has specialized systems such as receptors on the outer membrane and transport mechanisms to bring the iron into the bacterial cell [17,18,19]. These sophisticated strategies enable *Salmonella* to outcompete other gut bacteria for access to limited iron resources and give an advantage to thrive within the gut environment.

**Figure 1:**
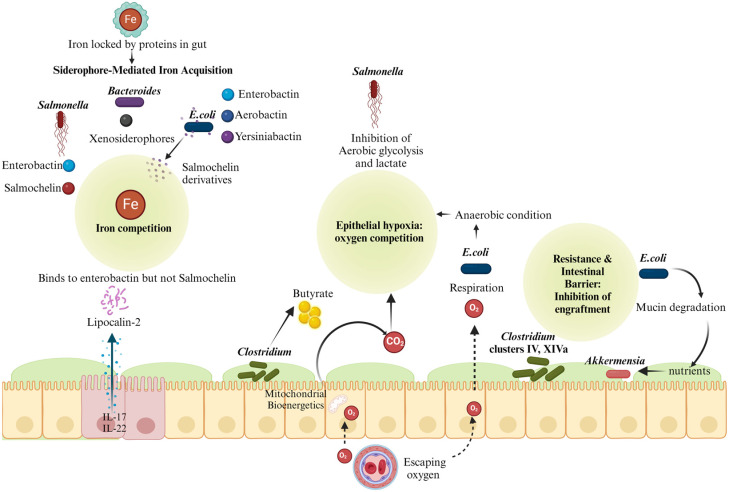
Schematic representation of ecocompetition (competition for resources) between commensal bacteria and *Salmonella* for survival in the gut. Gut bacteria and *Salmonella* compete for various nutrient niches in the gut for survival. This figure illustrates bacterial competition in the gut for iron and oxygen, and engraftment to intestinal cells. Bacteria like *E. coli* and *Bacteroides* produce siderophores to acquire iron, competing with salmochelin produced by *Salmonella*, which escapes Lipocalin-2, unlike other Enterobactins. Butyrate through *Clostridium* enhances mitochondrial bioenergetics, maintaining anaerobic conditions, supporting epithelial health, and limiting pathogenic bacterial colonization. *E. coli* consumes the remaining oxygen after *Clostridium*, creating anaerobic conditions that inhibit *Salmonella. E. coli* degrades mucin for nutrients, which creates nutrients for other bacteria like *Akkermensia* and other gut bacteria, making it harder for *Salmonella* to establish itself (created with http://www.BioRender.com).

*E. coli* and *Salmonella* belong to the same Enterobacteriaceae family, which produces the strongest siderophore enterobactin [20], allowing them to chelate ions from the environment. Following the induction of inflammation, IL-17 and IL-22 have been found to stimulate the intestinal lumen to produce lipocalin-2, a host antimicrobial protein [21]. Enterobactin, which Enterobacteriaceae also generates in the gut microbiota, binds to lipocalin-2, whereas salmochelin, produced by *Salmonella*, does not [22]. *E. coli* is bacteriostatically affected by the sequestration of enterobactin, but not salmochelin, which permits *Salmonella* to bloom in the lumen of the inflamed colon [22,23]. Commensal *E. coli* also produces aerobactin and yersiniabactin, which may explain its fitness in the GI tract and its competition with pathogens in the case of infections [24]. These may also be one of the reasons for the colonization of *E. coli* in the gut and their excess proliferation during dysbiosis (Figure 1). However, this commensal has an advantage over the pathogen as the probiotic *E. coli* strain Nissle 1917 releases salmochelin derivatives conjugated to antimicrobial peptides called microcins M and H47, which are absorbed by salmochelin uptake systems [25,26]. After the salmochelin uptake systems absorb the salmochelin derivatives conjugated to antimicrobial peptides, they can effectively target and inhibit the growth of *Salmonella* bacteria in the gut. During inflammation, *Bacteroides thetaiotaomicron* relies on xenosiderophores, which are siderophores produced by other bacteria, for iron acquisition. Specifically, *B. thetaiotaomicron* utilizes the XusABC system to access enterobactin and salmochelin, produced by different bacterial species. This mechanism is crucial for *B. thetaiotaomicron* colonization and gut survival during inflammation [27]. This also implies competition between commensal *E. coli* and *Bacteroides* for iron, and this competition serves as one of the regulatory mechanisms to prevent the unchecked proliferation of Enterobacterales like *E. coli*, which would otherwise be a prior signature of dysbiosis.

### 2.2. Oxygen: Epithelial Hypoxia

Epithelial hypoxia is a significant host-derived habitat filter that influences the variety of organisms living in the colon. The healthy colonic epithelium is always in a state of physiological hypoxia denoting <1% oxygen [28], which restricts the amount of oxygen that can diffuse into the colon’s lumen and maintain the anaerobic condition [29]. Oxygen is a critical resource for *Salmonella* to expand in the gut microbiota [30]. Aerobic respiration leads to the formation of lactate and formate, which is beneficial for the expansion of *Salmonella* [31,32]. Obligate anaerobic bacteria, therefore, control the microbial community, a phenotypic convergence in an important ecological characteristic. An increase in facultative anaerobic bacteria (*Enterobcaterales*) in the colonic microbiota [33] is significant for dysbiosis, which is caused by elevating epithelial oxygenation, which disturbs this biotic habitat filter due to an increase in oxygen availability in the intestinal lumen [34].

*Clostridia* in the large intestine helps break down fiber from our food into butyrate, which fuels the cells lining our intestines. This process uses up a lot of oxygen, making the area around these cells low in oxygen (hypoxic). Any leftover oxygen is quickly used up by Enterobacteriaceae members, which thrive in environments with a small quantity of oxygen (Figure 1). This synergy between *Clostridia* and Enterobacteriaceae such as *E. coli* keeps the gut mostly oxygen-free (anaerobic), which makes it harder for *Salmonella* to grow, reducing the risk of infection [30]. This cooperative working among the bacteria gives the gut microbiome its resilience capacity. If the synergy breaks either way, respiration will shift to aerobic respiration due to a decrease in either mitochondrial bioenergetics due to a decrease in butyrate or an accumulation of oxygen in the gut, which will increase in Enterobacteriaceae, leading to the disruption of further stability in the microbiome.

### 2.3. Resilience 

*Clostridium* can shape the gut environment by influencing its microbial composition. Although colonization resistance and resilience are associated with the entire gut microbiome and every gut microbe has synergy between them to maintain homeostasis, *Clostridium* clusters IV and XIVa are associated with a balanced and diverse gut microbiota [35]. This diversity contributes to competition for ecological niches and resources, potentially limiting the space and nutrients available for *Salmonella* within the gut ecosystem. SslE, a secreted and surface-associated lipoprotein from *E. coli*, serves several functions in the intestinal environment. Primarily, it contributes to the breakdown of mucin, the gel-like substance that lines the gastrointestinal tract, thus aiding in the degradation of the mucus layer [36]. This activity facilitates the shaping of the *E. coli* community within the intestine and promotes its long-lasting colonization (Figure 1). Additionally, by participating in mucin breakdown, SslE ensures the availability of essential nutrients that support the growth and survival of mucus-digesting bacteria such as *Akkermensia* and inhibits the engraftment of *Salmonella* on the epithelial lining during infection in the gut. *B. fragilis* also inhibits the translocation of *Salmonella* across the intestinal epithelial barrier, preventing systemic circulation and subsequent illness by the pathogen [37].

## 3. Metabolites

### 3.1. Bacteriocins

Nearly a century ago, the discovery of cationic, ribosomally produced antimicrobial peptides, called bacteriocins, marked a significant milestone. Their mechanism of action involves pore formation, the disruption of target cell membrane integrity, and, ultimately, cell death. Specifically, lactic acid bacteria are renowned for producing various bacteriocins characterized by non-toxicity, tolerance to heat and pH, and a broad spectrum of antimicrobial activity. These features render them ideal candidates for combatting infections [38]. 

Amylase is an enzyme that breaks down complex carbohydrates (starches) into simpler sugars (such as glucose and maltose) in the small intestine, providing a readily available carbon source that supports bacterial growth and metabolism and can help in the growth of *Lactobacillus* indirectly. Studies have shown that the presence of fermentable sugars enhances the proliferation of *Lactobacillus*, thereby potentially increasing their metabolic activities, including bacteriocin production. The improved availability of nutrients due to amylase activity can lead to a higher yield of bacteriocins, thus enhancing the antimicrobial capabilities of *Lactobacillus* cultures [39]. *Lacticaseibacillus casei* GG and *Lactobacillus johnsonii* La1 have shown antagonistic effects against *S.* Typhimurium by its complete expulsion [40,41]. *S. enterica* was inhibited by the combined action of nisin A and the *Lactobacillus acidophilus* surface layer protein [42]. Nisin A is a natural antimicrobial peptide primarily produced by *Lactococcus lactis*. It is commonly found in dairy products, particularly those that undergo fermentation using lactic acid bacteria. This suggests that while nisin A and the *L. acidophilus* surface layer protein may have antimicrobial properties individually, their combined action results in a more vigorous inhibition of *S. enterica*. Nisin A disrupts bacterial cell membranes, leading to cell death, while the surface layer protein of *L. acidophilus* may interfere with the adherence ability of *Salmonella* to intestinal epithelial cells or disrupt its membrane integrity through a different mechanism. Together, these compounds likely target multiple aspects of physiology, making it more difficult for the pathogen to survive and proliferate in the gut.

Historically, bacteriocins by *E. coli* have been categorized into two main groups: microcins and colicins. These bacteriocins exhibit over 30 distinct types, each characterized to varying degrees [43]. Probiotic strains such as EcNissle (*E. coli* Nissle 1917 and *E. coli* O6:K5:H1) and EcColinfant (*E. coli* A0 34/86 and *E. coli* O83:K24:H31) are utilized for human consumption [44]. *E. coli* release peptides known as MccB17 and MccJ25 microcins (Table 1). When there are few vital nutrients, microcins are generated and released and become active against related species. The FhuA-dependent TonB pathway and the outer-membrane protein OmpF, MccB17, and MccJ25 microcins internalize into the target bacterial cell through the outer-membrane receptor. Subsequently, both microcins are transported to the cytoplasmic face across the inner membrane by the inner-membrane protein SbmA. Ultimately, within the host cell, MccB17 functions as an inhibitor of DNA gyrase, and MccJ25 inhibits the bacterial RNA polymerase [45], inhibiting the growth of *Salmonella.*

### 3.2. Short-Chain Fatty Acids 

**Figure 2:**
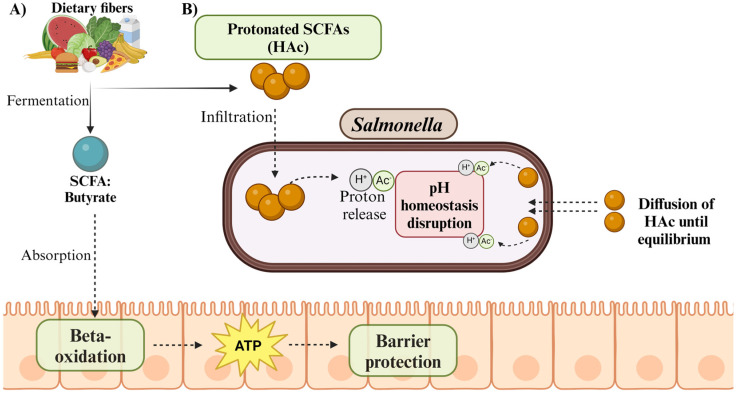
Schematic representation of fatty acids (SCFAs) inhibiting *Salmonella* in the gut. (**A**) Dietary fibers undergo fermentation, producing butyrate, an energy source for epithelial cells. It is crucial for maintaining the integrity and function of the colonic epithelial barrier and supporting other cellular processes essential for gut health. (**B**) Upon entry, the SCFAs release protons (H+), disrupting the internal pH homeostasis and causing acidification within the bacterial cell. The protonated SCFAs continue to enter until the intracellular pH matches the environmental pH. This internal pH imbalance inhibits *Salmonella* growth and survival (created with http://www.BioRender.com).

The importance of SCFAs in providing colonization resistance against *Salmonella* and other *Enterobacterales* has been highlighted by recent studies [38,46]. Acetate, propionate, and butyrate are SCFAs that prevent *Salmonella* from proliferating and colonizing in the gut environment. Protons (H+) are released when protonated SCFAs infiltrate *Salmonella*, upsetting the pH equilibrium inside the cell [47]. An ideal intracellular pH range is necessary for the synthesis of ATP by oxidative phosphorylation, which is disrupted in pH homeostasis. As a result, the development of *Salmonella* and metabolic activities are hampered. Furthermore, the luminal pH of the gut is changed by the presence of SCFAs, which makes the environment unsuitable for survival and growth [47,48]. Both the luminal pH and the concentration of SCFAs impact their inhibition against *Salmonella*; lowering pH values with greater SCFAs increases the antibacterial activity (Figure 2B). Through these pathways, SCFAs aid in preserving intestinal homeostasis and offer defense against *Salmonella* infection [49].

In addition to promoting epithelial hypoxia, butyrate production by *Clostridium* plays a crucial role against *Salmonella* in the gut through several mechanisms. Butyrate is a major energy source for colonocytes, promoting epithelial health and integrity (Table 1) [50]. When butyrate is produced through the fermentation of dietary fiber, it is absorbed by the colonic epithelial cells. Inside these cells, butyrate undergoes beta-oxidation, a process similar to fatty acid metabolism, to produce ATP. This energy is crucial for maintaining the integrity and function of the colonic epithelial barrier and supporting other cellular processes essential for gut health [50,51]. Thus, butyrate acts as a vital fuel source for the cells lining the colon, contributing to overall gastrointestinal function and homeostasis. This helps fortify the gut barrier, making it more difficult for *Salmonella* to invade the intestinal epithelium and establish infection (Figure 2A). In a mixed culture of *Lactobacillus crispatus* and *Anaerotignum lactatifermentans* on lactose, *L. crispatus* ferments lactose to lactate, and *A. lactatifermentans* ferments lactate to acetate and propionate. The growth of *S.* Typhimurium is inhibited by these undissociated forms of acetate and propionate produced in the mixed culture [52]. *Bacteroides* also produce propionate, which may be beneficial against *Salmonella* colonization as it has been found to have antimicrobial effects against multi-drug resistant (MDR) pathogens, including *Salmonella*, by disrupting intracellular pH homeostasis [53,54]. SCFA butyrate, acetate, and propionate effectively limit *Salmonella* motility, biofilm formation, and gene expression [55]. 

### 3.3. Vitamins

Vitamins are essential for the normal functioning of the human body. They are obtained from diet intake, or certain commensal gut bacteria can also produce them. Bacteria in the gut produce riboflavin (VB2), pyridoxine (VB6), and folacin (VB9), called the B vitamins [56]. Cellular and humoral immunity depends on VB6, produced by bacteria such as *E. coli*. Low VB6 levels compromise immunological responses. *Bacteroides acidifaciens* and VB6 supplementation have demonstrated potential in improving pathogen clearance after *S.* Typhimurium infection. The synergy between *B. acidifaciens* and VB6 supplementation in improving pathogen clearance after *S.* Typhimurium infection likely arises from multiple factors (Table 1). First, VB6 serves as a crucial cofactor for various metabolic pathways in *B. acidifaciens*, promoting its growth and metabolic activity [57]. As *B. acidifaciens* flourishes in the gut environment due to VB6 supplementation, it exerts regulatory effects on the host immune system, enhancing its ability to fight against *Salmonella* either by modulating immune responses through interactions with immune cells or by influencing the production of immune-regulatory molecules. Secondly, *B. acidifaciens* might also interfere with *Salmonella* virulence factors directly or indirectly, either by competing for resources or by producing antimicrobial compounds that may inhibit *Salmonella* growth (Figure 1, Table 1).

**Table 1:** Mechanism of action by different commensal bacteria against *Salmonella* in gut.

Bacteria	Metabolites		Mechanism of Action	References
*Clostridium*	SCFA	Butyrate	Energy source for colonocytes	[50]
			pH homeostasis	[10]
*Lactobacillus*	Bacteriocins	Nisin A	Antimicrobial action through disruption of membrane	[40,41]
	Nisin A + S layer protein			[42]
*Bacteroides*	SCFA	Propionate	Disrupting intracellular pH homeostasis	[54]
	Vitamin	VB6	Enhances *Bacteroides* growth	[57]
*E. coli*	Microcins	MccB17 MccJ25	Targeting bacterial nucleic acid	[45]

## 4. Immune Modulation

Unlike other pathogenic bacteria, *Salmonella* modulates the host immune system and cell signaling in favor of its expansion in the gut. The subversion of host immune cells, especially macrophages, which are essential for pathogen defense, is one important way. When macrophages encounter *Salmonella*, they ingest the bacterium in a phagocytosis process to start an immunological response. However, *Salmonella* has developed extreme ways to live and spread inside macrophages, effectively taking control of these defense cells and using them as holding tanks for the growth of the bacteria, such as delayed cytotoxicity, which inhibits the inflammatory response against this pathogen [58]. *Salmonella* manipulates intracellular signaling pathways and evades host immune responses by delivering effector proteins directly into host cells through specific virulence factors, such as type III secretion systems (T3SS) [6]. *Salmonella* effectors, for instance, can obstruct phagosome maturation, stopping *Salmonella*-containing vacuoles from merging with lysosomes and protecting the germs from lysosomal enzyme destruction. *Salmonella* effector proteins can also alter the cytoskeletal dynamics of host cells, which encourages bacterial uptake and intracellular survival. In addition, *Salmonella* has the ability to cause inflammation in the mucosa of the stomach, attracting immune cells and impairing the function of the epithelial barrier, all of which promote bacterial translocation and systemic spread [59,60]. Subsequent to *Salmonella* infiltration, M2 macrophages are triggered. These macrophages, in turn, upregulate anti-inflammatory cytokines such as interleukin-10 (IL-10) and downregulate the proinflammatory cytokines such as IL-6 and tumor necrosis factor (TNF-α) [61], which helps in the spread of the infection. Such modulation aids in the propagation of the infection. By using these complex mechanisms, *Salmonella* evades the host immune response and fosters an environment within the gut to proliferate and colonize, eventually leading to infection and disease. 

### 4.1. Proinflammatory Cytokine Stimulation

**Figure 3:**
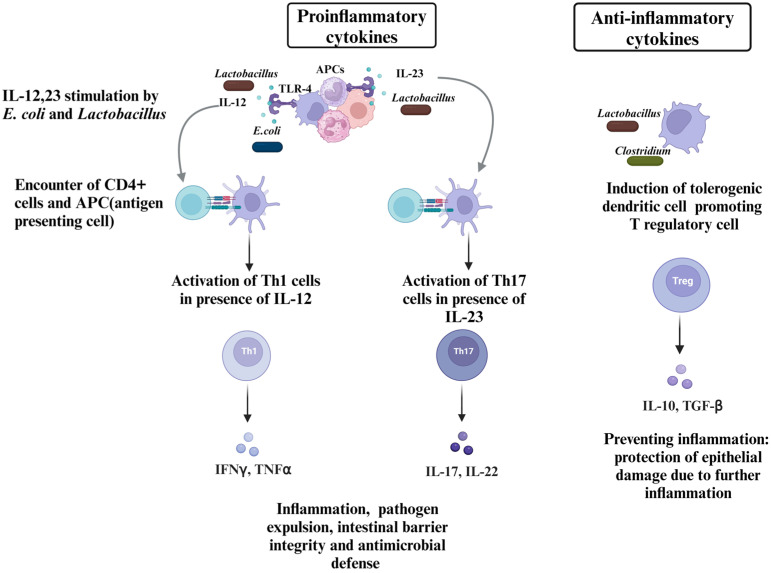
Schematic representation of immune modulation by commensal bacteria against *Salmonella* in the gut. Non-pathogenic *E. coli* and *Lactobacillus* stimulate APCs through IL-12 and TLR-4 activation. This leads to the activation of CD4+ T cells and their differentiation into Th1 cells in the presence of IL-12. Th1 cells produce IFN-γ and TNF-α, promoting inflammation, pathogen expulsion, intestinal barrier integrity, and antimicrobial defense. Additionally, *E. coli* and *Lactobacillus* stimulate APCs to produce IL-23, inducing the differentiation of CD4+ T cells into Th17 cells. Th17 cells secrete IL-17 and IL-22, enhancing gut barrier integrity by increasing tight junction protein expression and mucin production. On the other hand, *Lactobacillus* and *Clostridium* promote the development of tolerogenic dendritic cells, which induce Treg cells. Treg cells secrete IL-10 and TGF-β, suppressing excessive inflammation, protecting epithelial cells from damage, and maintaining gut homeostasis. These interactions highlight the complex role of gut microbiota in modulating immune responses, balancing proinflammatory and anti-inflammatory signals to protect against *Salmonella* infection while preventing excessive tissue damage (created with http://www.BioRender.com).

Evidence suggests that non-pathogenic *E. coli* strains, such as 129 and 13-7, influence the host immune system through the stimulation of IL-12 and Toll-like receptor-4 (TLR-4) [62]. This pathway indicates an activation of the innate immune response, which plays a crucial role in recognizing and responding to *Salmonella* (Figure 3). Additionally, the dominant *E. coli* strains such as *E. coli* 083: K24:H31 colonized in the infant guts correlate with heightened local serum and antibody responses (IgA and IgM), suggesting a potential role in adaptive immune system activation [63]. The activation of TLR-4 and IL-12 by *E. coli* is beneficial for protection through several mechanisms. First, TLR-4 activation causes the release of chemokines and proinflammatory cytokines, which attract and strengthen immune cells near the infection site. This aids in limiting the spread of *Salmonella* and leads to early detection. Also, TLR-4 activates antimicrobial peptides (AMPs), such as defensins, and cathelicidins, preventing *Salmonella* from growing [64]. Second, T cells are encouraged by IL-12 to differentiate into T-helper cells (Th1 cells), which are responsible for cell-mediated protection against *Salmonella*. Th1 cells activate cytotoxic T lymphocytes and macrophages to destroy infected cells [65,66]. Furthermore, CD8+ T cells and natural killer (NK) cells, essential in preventing *Salmonella* reproduction and dissemination, become more cytotoxic by IL-12 by further differentiation and proliferation [67].

The IL-23/IL-22 and IL-23/IL-17 pathways play crucial roles in maintaining gut barrier integrity and protecting against *Salmonella* infection (Figure 3). IL-22, primarily produced by immune cells like T cells and innate lymphoid cells, acts on epithelial cells to enhance the expression of tight junction proteins, such as claudins and occludins, thereby reinforcing the barrier between intestinal epithelial cells. Additionally, IL-22 stimulates the production of mucins, which form a protective layer over the epithelium, and antimicrobial peptides, which help to control the growth of *Salmonella*. IL-17, mainly secreted by Th17 cells, also contributes to barrier defense by promoting the secretion of mucins and enhancing the expression of tight junction proteins [68]. *Bacteroides*-produced PSA (zwitterionic-polysaccharide) impacts the development of CD4+ T cells and modulates the immune equilibrium between T-helper cell subsets (Th1/Th2) [69]. These actions strengthen the gut barrier, reducing the risk of *Salmonella* invasion and maintaining intestinal permeability. This barrier serves as a selective means of preventing pathogens, nutrients, and water from entering the bloodstream through the gut lumen [70]. The intestinal barrier is weakened during *Salmonella* infection due to various processes the pathogen orchestrates. *Salmonella* increases the permeability of the intestinal epithelium because it damages the tight junctions essential to preserving the integrity of the epithelial barrier [59]. Certain virulence factors expressed by *Salmonella* pathogenicity islands, such as effectors supplied by T3SS, enhance this disruption [59]. Moreover, *Salmonella* actively harms the mucosa of the stomach by secreting virulence factors and toxins like lipopolysaccharide (LPS) and cytotoxic effectors delivered by T3SS, which cause sloughing and destruction to the epithelium cells. *Lactiplantibacillus plantarum* ZS2058 and *Lacticaseibacillus rhamnosus* GG enhance protective mechanisms by modulating immune responses and promoting the production and action of IL-22 and IL-17, thus enhancing gut health and host defense against pathogens [71,72]. Furthermore, both ZS2058 and LGG have been found to restore the levels of IFN-γ, a cytokine suppressed by *Salmonella* [71]. 

### 4.2. Anti-Inflammatory Cytokine Stimulation

Regulatory T cells (Tregs) play a critical role in immune regulation by suppressing excessive immune responses and maintaining tolerance to self-antigens and harmless foreign substances (Figure 3). One of the mechanisms through which Tregs exert their regulatory function is by secreting anti-inflammatory cytokines, such as IL-10. IL-10 is known for its potent anti-inflammatory properties, as it inhibits the production of proinflammatory cytokines and downregulates the immune cell activity in inflammation. Beyond metabolic interactions, *Clostridium* promotes Treg cell development [73]. *Bacteroides* also activate the IL-10 [69]. *Lactobacillus* has also been found to regulate inflammation by changing TNF-α, IL-10, and myeloperoxidase (MPO) levels [71] and superoxide dismutase-producing *L. casei* BL23, neutralizing reactive oxygen species (ROS) [74]. *A. muciniphila* also employs mechanisms that are pivotal in orchestrating the distinct promotion of Treg cells within the gut microenvironment as its deficiency causes a decline in the Treg cells [75] making this bacteria important for immunomodulation. These regulatory mechanisms prevent excessive inflammation in the gut, which can lead to tissue damage and exacerbate the severity of the infection. By promoting Treg development and IL-10 production, the immune system can effectively control *Salmonella*-induced inflammation while minimizing collateral tissue damage, thereby facilitating the resolution of the infection and promoting overall gut health. Even in the absence of a *Salmonella* infection, commensal bacteria in the gut can, under normal circumstances, interact with the host immune system and cause the activation of immune cells. However, this may be a part of the intricate immune surveillance system in the gut. Pattern recognition receptors (PRRs) on immune cells, such as TLR-4, bind to commensal bacteria, triggering low-level immune response activation which prepares the gut for an unexpected pathogen intervention, and the anti-inflammatory signaling involving Treg cells can balance those basal immune responses or protect the gut lining in case of excess inflammation by proinflammatory cytokines. 

## 5. External Factors

The gut microbiome is not just the microbiota but a combination of host-derived habitat filters, microbiota-nourishing community, microbial resistance, and environmental factors, mainly host diet behaviors [10]. The host diet patterns also change the species richness and diversity based on what needs to be broken down by the microbes in our gut, in turn increasing their activity in the gut.

### 5.1. Effect of Diet

Dietary fat intake can increase primary bile acid concentrations within the intestinal lumen, promoting the colonization of *S.* Typhimurium [76], and *Salmonella* exhibits adaptations to thrive in a bile-rich environment [77], including the upregulation of virulence genes [78], rendering them more resistant to bile. These survival strategies include efflux pumps and outer membrane proteins, the modification of lipopolysaccharide and membrane structures, and the induction of virulence factors [79]. In the gut, fibers are fermented by *Clostridium* and other commensal bacteria, producing SCFAs. SCFAs function as signaling molecules that regulate immunological responses by binding to specific receptors on immune cells. SCFAs can attach to intestinal epithelial cells and immunological cells, such as Tregs and dendritic cells, via G-protein-coupled receptors (GPCRs) like GPR41 and GPR43. Proinflammatory cytokines are inhibited by this interaction, whereas anti-inflammatory cytokines like IL-10 and transforming growth factor-beta (TGF-beta) are secreted in greater amounts [80]. Furthermore, SCFAs can differentiate tolerogenic dendritic cells, which promotes Treg production and proliferation [80]. Thus, it shows how important diet is in the case of *Salmonella*-induced infection as a high-fat diet poses a higher risk for *Salmonella* infections and their sustainability due to their adaptation to the bile environment in the gut. Excessive fat intake may alter the gut microbiome composition, reducing beneficial bacteria and promoting pathogenic ones. This imbalance can weaken the gut barrier and immune response, making it easier for *Salmonella* to thrive. High fat levels may also lead to increased inflammation and the disruption of gut pH, further facilitating *Salmonella* survival and virulence. 

### 5.2. Antibiotics

Antibiotics such as fluoroquinolones (e.g., ciprofloxacin) and third-generation cephalosporins (e.g., ceftriaxone) have been used to target specific *Salmonella* strains in systemic infection [81]. However, antibiotics must be used cautiously, as their overuse can contribute to the development of antibiotic resistance. Consequently, beneficial bacteria that typically provide colonization resistance against *Salmonella* may be suppressed, allowing the pathogen to thrive unchecked. Natural compounds exhibit anti-*Salmonella* properties, reducing growth and inflammation in the gut. Quercetin, a flavonoid found in a variety of fruits and vegetables, has antimicrobial properties against *Salmonella* and inhibits its virulence factors [82]. Curcumin, a turmeric derivative, has anti-inflammatory and antibacterial properties, making it a potential treatment for *Salmonella* infections [83]. However, antibiotic treatment for intestinal *Salmonella* infections can present difficulties and hazards that affect the pathogen and the gut flora [84]. 

A chronic or persistent infection that necessitates lengthy or repeated courses of antibiotics may arise when antibiotic therapy cannot eliminate *Salmonella* from the stomach [85]. As a result, although antibiotics are still an essential tool for treating severe *Salmonella* infections, their use should be carefully considered. Other options may be investigated to reduce antibiotic treatment risks and maintain gut health, such as probiotics or targeted antimicrobial therapy [86]. 

## 6. Discussion

Gut microorganisms fight *Salmonella* through a variety of mechanisms. However, *Salmonella* has developed several strategies to overcome this barrier, which explains why the infection persists for years in the gut of afflicted individuals. Unlike other harmful bacteria, *Salmonella* employs a special method to colonize the gut microbiota. The trickiest means of survival for *Salmonella* is its abundance inside macrophages [87]. Changes in these areas result in altered mitochondrial bioenergetics, a rise in reactive species, altered microbial communities that compromise the intestinal barrier, the synthesis of SCFA, which inhibits colonization resistance, altered niche preemption, and a breakdown of the resilience of the microbiota. In addition, chronic dysbiosis conditions might emerge as an alternative equilibrium state if the microbiota cannot recover from the disturbance and several beneficial species become extinct [10]. Apart from T3SS, which *Salmonella* uses to enter the monocytes, upon inflammation, there is an increase in bile acid which activates T6SS. Induction of T6SS leads to the death of commensal bacteria in the gut by interbacterial competition, such as *Klebsiella oxytoca* [88]. 

The competition for development, inhibition through metabolites, protection of the intestinal barrier, immune system response, and external stimulation from the environment are the most crucial factors in cases of *Salmonella* infection in the gut. Each commensal bacterium in the gut performs a distinct role, and the pathogen must be eliminated for the commensal bacteria to work together harmoniously. Enterobacteriaceae and butyrate-producing bacteria must work together to maintain epithelial hypoxia [30]. Among the first bacteria to live in a newborn’s intestines are *Enterobacterales*, such as *E. coli* [89]. The harmony is also explainable by the fact that during vaginal delivery, *E.coli*, *Staphylococcus* spp., and *Streptococcus* spp. take over the gut initially to create an environment optimal for the *Bacteroides* spp. and *Bifidobacterium* spp. [90]. 

The morphology and physiological characteristics of the gastrointestinal system differ between humans and mice. For instance, the two species differ regarding the gut’s length, transit time, and distribution of its segments. These differences affect microbial colonization patterns, host–microbiota interactions, and nutrient absorption. Significant distinctions exist in the specific composition of the microbial communities found in the intestines of mice and humans, even though these populations are both diverse. An in-depth study of Yorkshire pig gut microbiota revealed similar diversity patterns to humans, with Bacteroidetes and Firmicutes as the dominant phyla. However, pigs had more *Spirochaetes* and *Prevotella* at the genus level compared to humans. These changes may impact the ability of the microbiota to operate and interact with the host [91]. This also creates an issue of translation between results from animal models to human applications. Therefore, in vitro studies using human gut microbiota are needed for proper understanding, confirmation, and application.

Understanding the intricate interplay between *Salmonella* and gut microbes is critical for developing effective prevention and treatment strategies for salmonellosis. Severe consequences may occur if the gut microbiota fails to protect the gut from *Salmonella*. *Salmonella* can take advantage of disruptions in the gut microbiota by factors such as antibiotic use, stress, or dietary changes to gain a competitive advantage, resulting in pathogen overgrowth [10]. This dysbiosis can cause a breach in the intestinal epithelial barrier, allowing *Salmonella* to invade host tissues and cause inflammation and systemic infection [8]. 

Future research on the complex interplay between *Salmonella* and gut microbes promises significant advancements in human health. Strategies for treating or preventing *Salmonella* infections include targeted therapies that alter the gut microbiota, dietary modifications, and probiotics. Other exciting avenues for research include investigating the gut virome and the function of protozoa in microbiota regulation. Additionally, studies on the potential of phytochemicals and natural compounds in regulating *Salmonella* infections and their interactions with the gut microbiota may lead to the development of novel therapeutic strategies. A more profound comprehension of these relationships will open the door to tailored interventions, enhancing our capacity to treat and avoid *Salmonella*-related health issues.

## Data Availability

No new data were created or analyzed in this study. Data sharing is not applicable to this article.

## List of Abbreviations

**AMPs**	Antimicrobial peptides
**ATP**	Adenosine Triphosphate
**GI**	Gastrointestinal tract
**GPCRs**	G-protein-coupled receptors
**IFN**	Interferon
**IgA, M**	Immunoglobulin A, M
**IL**	Interleukin
**LAB**	Lactic Acid Bacteria
**LGG**	*Lactobacillus rhamnosus* GG
**LPS**	Lipopolysaccharides
**MDR**	Multi-Drug Resistance
**MPO**	Myeloperoxidase
**NK**	Natural killer (cells)
**PRRs**	Pattern recognition receptors
**PSA**	Polysaccharide A
**ROS**	Reactive oxygen species
**SCFA**	Short-chain fatty acid
**SsIE**	Secreted and surface-associated lipoprotein from *E. coli*
**T3SS**	Type III secretion system
**T6SS**	Type VI secretion system
**TGF**	Transforming growth factor
**Th**	T-helper cells
**TLR**	Toll-like receptor
**TNF**	Tumor necrosis factor
**Tregs**	Regulatory T cells
**VB2,6,9**	Vitamins B2 (Riboflavin), B6 (Pyridoxine), B9 (Folate)
